# The evaluation of juvenile ocular hypertension by optical coherence tomography angiography

**DOI:** 10.1186/s12886-020-01641-4

**Published:** 2020-10-21

**Authors:** Xiaoxiao Chen, Xiaolei Wang, Xinxin Hu, Xinghuai Sun

**Affiliations:** 1 Department of Ophthalmology & Visual Science, Eye & ENT Hospital, Shanghai Medical College, Fudan University, Fenyang Road. 83, Shanghai, 200031 China; 2 State Key Laboratory of Medical Neurobiology, Institutes of Brain Science and Collaborative Innovation Center for Brain Science, Fudan University, Shanghai, China; 3 NHC Key Laboratory of Myopia (Fudan University), Key Laboratory of Myopia, Chinese Academy of Medical Sciences, and Shanghai Key Laboratory of Visual Impairment and Restoration, Fudan University, Shanghai, China

**Keywords:** Intraocular pressure, Cup/disc ratio, Juvenile ocular hypertension, Optical coherence tomography angiography, Vessel density

## Abstract

**Background:**

Vessel density (VD) of the elderly ocular hypertension patients measured by optical coherence tomography angiography (OCTA) have been reported. However, the studies of VD in juvenile ocular hypertension (JOHT) are limited. We wished to evaluate VD changes using OCTA in JOHT. We also investigated the potential risk parameters of intraocular pressure (IOP) and vertical cup/disc ratio (CDR) with OCTA for observing the development of JOHT.

**Methods:**

We examined 86 eyes in 45 control (Ctrl) subjects and 65 eyes in 34 patients with JOHT using OCTA at the glaucoma clinic of the Eye, Ear, Nose, and Throat Hospital of Fudan University. The VD of radial peripapillary capillaries (RPC) and the perifoveal superficial vascular plexus (SVP) was compared between the Ctrl and JOHT groups. Other basic study factors such as age, sex, blood pressure, best-corrected visual acuity, central corneal thickness, IOP, CDR, the thickness of the retinal nerve fiber layer, ganglion cell complex, visual field mean deviation, and pattern standard deviation were also recorded.

**Results:**

Bare difference was found in the nasal-inferior and temporal RPC-VD between the Ctrl and JOHT groups (*P* = 0.042 and *P* = 0.033, respectively) while SVP-VD was not (all *P* > 0.05). In the mixed linear regression model analysis, temporal RPC-VD was marginally negatively associated with high IOP (r = − 1.379, *P* = 0.043). Five additional sections of nasal, inferior-nasal, inferior-temporal, superior-temporal, and superior-nasal RPC-VD showed positive correlation with large CDR (all *P* <  0.05). SVP-VD in the superior and nasal regions was marginally negatively correlated with high IOP (r = − 1.877, *P* = 0.023; r = − 1.693, *P* = 0.049). No other regions were found statistical different of relationship between IOP, CDR and VD.

**Conclusions:**

Nasal-inferior and temporal peripapillary VD was marginally lower in JOHT subjects. Regarding parameters of IOP and CDR, peripapillary temporal VD had a borderline level of negative correlation with IOP more than 21 mmHg while additional five regions were strongly positively correlated with large CDR. Some macular regions only found marginal positive correlation with parameter of high IOP. We conclude that OCTA can be used as a potential technique to evaluate the VD in JOHT and peripapillary scans should be analyzed individually based on different levels of CDR.

## Background

Ocular hypertension (OHT) is defined as intraocular pressure (IOP) higher than 21 mmHg with normal optic disc structure/function and no previous history of angle closure. Approximately 5% of OHT patients develop glaucoma after 5 to 7 years in follow-ups [1]. Glaucoma is the leading cause of irreversible blindness, including both adult-onset disease (occurring after 40 years of age) and juvenile-onset disease (occurring between the ages of 3 and 40); the IOP of juvenile-onset patients is often extremely high, with a more aggressive clinical course [2, 3]. Thus, OHT in juveniles (JOHT) should be given more attention when considering long-term follow-up. However, JOHT presents few ocular symptoms and little disturbance of visual acuity, making its diagnosis and management a difficult clinical challenge [4]. In addition, previous literature shows that the normal range of IOP in juveniles may be different from that in adults [5]. Currently, there is no consensus in the literature that offers guidance to the clinician in determining when and whether JOHT should be treated. Evidence provided by previous studies concerning OHT mainly focuses on people more than 40 years old under traditional diagnostic standards [1]. The Ocular Hypertension Treatment Study (OHTS) established that medically treating ocular hypertension is efficacious in delaying or preventing the onset of glaucoma, while the European Glaucoma Prevention Study (EGPS) failed to demonstrate the significance of reducing IOP in preventing the onset of the disease [6, 7]. The effects of lowering IOP in JOHT subjects were ambiguous. Moreover, JOHT individuals are increasingly being identified through the prevalence of optical screening, and some of them even demonstrate a large vertical cup/disc ratio (CDR). This is considered a good qualitative predictor for the onset of glaucoma in JOHT, although this correlation has not been empirically verified [8]. The CDR index has long been used in the assessment of JOHT, though the wide range of CDR values in the normal population from 0.00 to 0.87 limits its utility [9, 10]. Thus, decisions regarding the therapy of JOHT can be difficult, especially for those with large CDR [11, 12]. New modalities should be considered to assess JOHT patients and find how the predictors of CDR and IOP impact the evaluation of the disease.

Previous experimental and clinical investigations have provided evidence showing a strong correlation between vascular dysregulation and glaucoma [13, 14]. Recently, the rapidly evolving technology of optical coherence tomography angiography (OCTA) has been utilized to measure local retinal circulation without the need for dye infusion. The split-spectrum amplitude-decorrelation angiography (SSADA) algorithm can be used to quantify blood flow [15]. Radial peripapillary capillaries (RPC) comprise a network of capillary beds located within the retinal nerve fiber layer (RNFL) that supplies the retinal ganglion cell (RGC) axons; damage to RNFL and RGC are typical manifestations of glaucoma [16]. Thus, OCTA could quantitatively characterize the microvasculature around the nerve head to find the preclinical vessel density (VD) changes of JOHT. The IOP of JOHT typically remains fluctuated, especially in subjects more than 10 years old [4]. No typical clinical symptoms or pathological changes can explain the relationship between IOP and retinal perfusion [4]. Previous studies using OCTA have shown that PRC-VD is significantly reduced in glaucoma and related to the severity of the disease [17, 18]. Additionally, the macular VD of the superficial retinal vascular plexus (SVP) has also been investigated, and a reduction in macula has been found [19]. It is valuable to study the peripapillary and macular VD profile of JOHT and to find the relationship between IOP and perfusion. Besides, variation in CDR requires more accurate and quantatitive assessment in evaluating the peripapillary microvasculature. Therefore, investigating the use of non-invasive and high-resolution OCTA techniques for JOHT subjects is an important research avenue to pursue.

The purpose of this study was to evaluate RPC-VD and SVP-VD in JOHT using OCTA and to examine the relevance of IOP and CDR to the VD profile.

## Methods

### Study participants

This was a cross-sectional study. Participants were recruited from August 1, 2017 to July 1, 2018 at the glaucoma clinic of the Eye, Ear, Nose, and Throat Hospital of Fudan University in Shanghai, China. The study received approval from the Ethical Review Committee of the Eye, Ear, Nose, and Throat Hospital and adhered to the tenets of the Declaration of Helsinki. Each participant provided written informed consent before examination.

Eligibility was determined by a complete ophthalmologic examination, including slit-lamp biomicroscopy, best-corrected visual acuity (BCVA), refractive error (RE) measured with an autorefractor (KR-890; Topcon, Tokyo, Japan), IOP measurement with Goldmann applanation tonometry, gonioscopy, axial length (AL) and central corneal thickness (CCT), A-scan ultrasound (A-scan Pachymeter, Ultrasonic, Exton, PA, USA), and dilated fundus examination. Standard automated perimetry (SAP) (30–2 Swedish Interactive Threshold Algorithm; Humphrey Field Analyzer II; Cal Zeiss Meditec, Inc., Dublin, CA), spectral-domain optical coherence tomography (SD-OCT) (RTvue OCT; Optovue Inc., Toledo, OH), and OCTA (RTVue-XR Avanti, software version 2014.2.0.65; Optovue, Inc.; Fremont, CA, USA) were also accomplished by operators. OCTA scans were repeated at least twice, and the mean of the results was evolved in the final analysis. Systematic information, including age, sex, blood pressure (BP), and pulse rate (PR), were collected. BP was recorded as systolic blood pressure (SBP) and diastolic blood pressure (DBP). The mean arterial pressure (MAP) was calculated as the following formula: MAP = 2/3DBP + 1/3SBP. The mean ocular perfusion pressure (MOPP) was calculated by subtracting the IOP from two-thirds of the MAP [20].

Inclusion criteria for all subjects were: (1) age > 3 years old and < 40 years old, (2) normal open-angles on gonioscopy, (2) BCVA > = 12/20, (3) AL < 26.50 mm and RE < − 6.0D, (4) average RNFL and ganglion cell complex (GCC) thickness within 99% confidence limits, (5) a minimum of two reliable normal visual fields (i.e., false-positive errors < 15%, false-negative errors < 15%, and fixation loss < 20%), defined as a pattern standard deviation (PSD) and mean deviation (MD) within 95% coincidence limits within 6 months. Exclusion criteria for all groups were previous intraocular surgeries, nonglaucomatous optic neuropathy, retinopathies or secondary elevated IOP and other ocular diseases. Participants were also excluded if there was a diagnosis of hypothyroidism, diabetes mellitus, cardiovascular diseases, or abnormal hemorheology [21].

Major inclusion criteria of control subjects (Ctrl) featured normal optic disc accord with ISNT rule, intact neuroretinal rim, and IOP < = 21 mmHg with no history of elevated IOP. JOHT is with untreated IOP of > 21 mmHg and < 32 mmHg [1].

### OCTA image acquisition and processing

All the OCTA scans were acquired via a commercial spectral domain system. The system uses an A-scan rate of 70 kHz and has a light source centered on 840 nm and a bandwidth of 45 nm. Both eyes of each participant were operated on and scanned within the same visit. The uses of two repeated B-scans at 304 raster positions allowed the acquisition of three-dimensional (3D) OCTA scans, with each B-scan consisting of 216 A-scans. With a B-scan frame rate of 210 frames per second, each OCTA volume scan could be acquired in approximately 3 s. An en face retinal angiogram was created by projecting the flow signal internally to the retinal pigment epithelium. All this processing was achieved using the software included above. Motion artifacts were removed by 3D orthogonal registration and merging the two scans. SSADA was described in previous publications and developed to overcome the pulsatile bulk motion noise in the axial direction [16].

**Fig. 1:**
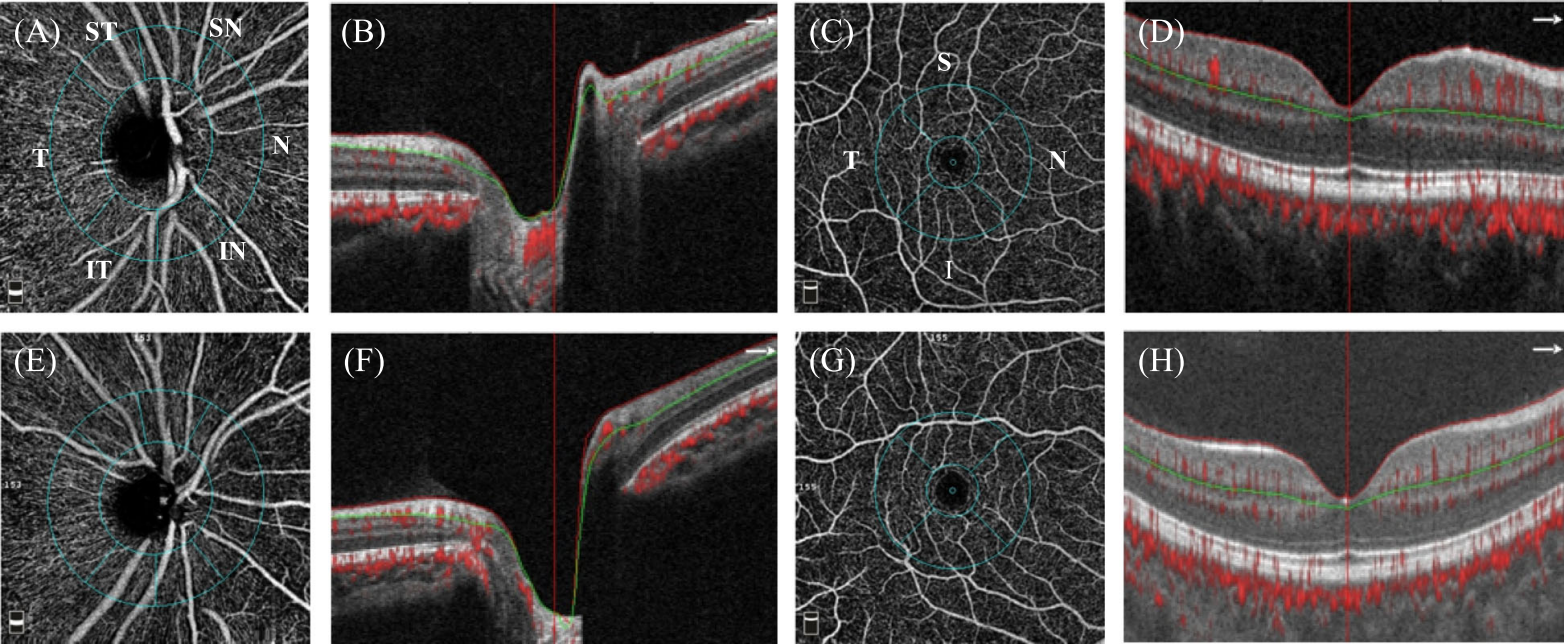
Measurement points of optical coherence tomography angiography (OCTA) scans. **a**-**d** OCTA scans of control subjects. **a** Peripapillary retinal microvasculature centers on the nerve head. The ring-shape region of interest is divided into six parts automatically as nasal (N), inferior nasal (IN), inferior temporal (IT), temporal (T), superior temporal (ST), and superior nasal (SN). Signals inside the inner circle are defined as vessel density of the inside disc. Pixels of large blood vessels will be excluded during analysis. **b** The boundaries used for segmentation are indicated by the green line (inner plexiform layer, IPL) and the red line (inner limiting membrane, ILM). The radial parapapillary capillaries (RPC) from ILM to the retinal nerve fiber layer (RNFL) is analyzed in the scans of the nerve head. The arrow represents the nasal direction. **c** Perifoveal retinal microvasculature centers on the macula showing the superficial retinal vascular plexus (SVP). The area inside the inner circle, including the foveal avascular zone (FAZ), is excluded from the analysis. The ring-shaped region of interest is automatically separated as hemi-superior (Hemi-S), hemi-inferior (Hemi-I), nasal (N), inferior (I), tempo (T), and superior (S). **d** SVP from ILM to IPL is analyzed. The red line and the green line possessed the same definition as the lines in image (**b**). **e**-**h** Images of juvenile ocular hypertension (JOHT) subjects

OCTA was used to quantify the VD of both the optic disc and the macula. The peripapillary and perifoveal VD were calculated using the proportion of the measured area occupied by flowing blood vessels as the pixels with decorrelation values over the threshold in SSADA. Scans were obtained over a 4.5 × 4.5 mm region centered at the optic nerve head and a 6.0 × 6.0 mm region centered at the fovea (Fig. 1). Retinal layer segmentation was analyzed automatically to segment the inner limiting membrane (ILM) and the outer boundary of the inner plexiform layer (IPL) for both peripapillary and perifoveal scans (Fig. 1b, d, f, h). RPC plexus from ILM to RNFL for peripapillary scans and SVP from ILM to IPL for perifoveal scans were recorded and analyzed, respectively. The peripapillary region was defined as a 700 μm wide elliptical extending outward from the boundary of the optic disc. The peripapillary area was divided into six sectors, as designated by Garway-Heath (Fig. 1a, e) (N, nasal; I, inferior; T, temporal; S, superior) [22]. The perifoveal retinal perfusion was measured using a masking procedure. The masking overlay consisted of an annulus, defined by an inner diameter of 0.6 mm and an outer diameter of 6 mm, and the region was divided into four sectors according to the ETDRS regionalization (Fig. 1c, g). The large vessels were excluded from the image to calculate only the capillary density, while scans with a low signal strength index of less than 48 or motion artifacts were also excluded [23].

### Statistical analysis

Patients’ characteristics between two groups were compared using a two-sample t-test or Kruskal-Wallis rank sum test depending on whether the data were in accord with Gaussian distribution or not. Continuous variables are described as means ± standard deviation (SD). A chi-square test was used to analyze the frequency data on gender. Both of the eyes of participants up to inclusion criteria were selected for the analysis; thus, a linear mixed model was used to detect the differences in eye-level covariates between Ctrl and JOHT due to within-subject correlation. Parameters of IOP > 21 mmHg or CDR > 0.6 were given the value of 1, while the opposite was assigned to 0 to assess the impact of high IOP and large CDR on VD using a linear mixed model. As FD-OCT showed a higher sensitivity and lesser specificity in calculation of CDR, a ratio slightly higher than ophthalmoscopic standards was applied [24]. All analyses were performed by SAS software (v 9.4, Inc., Cary, NC). A *p*-value less than 0.05 was considered statistically significant.

## Results

**Fig. 2:**
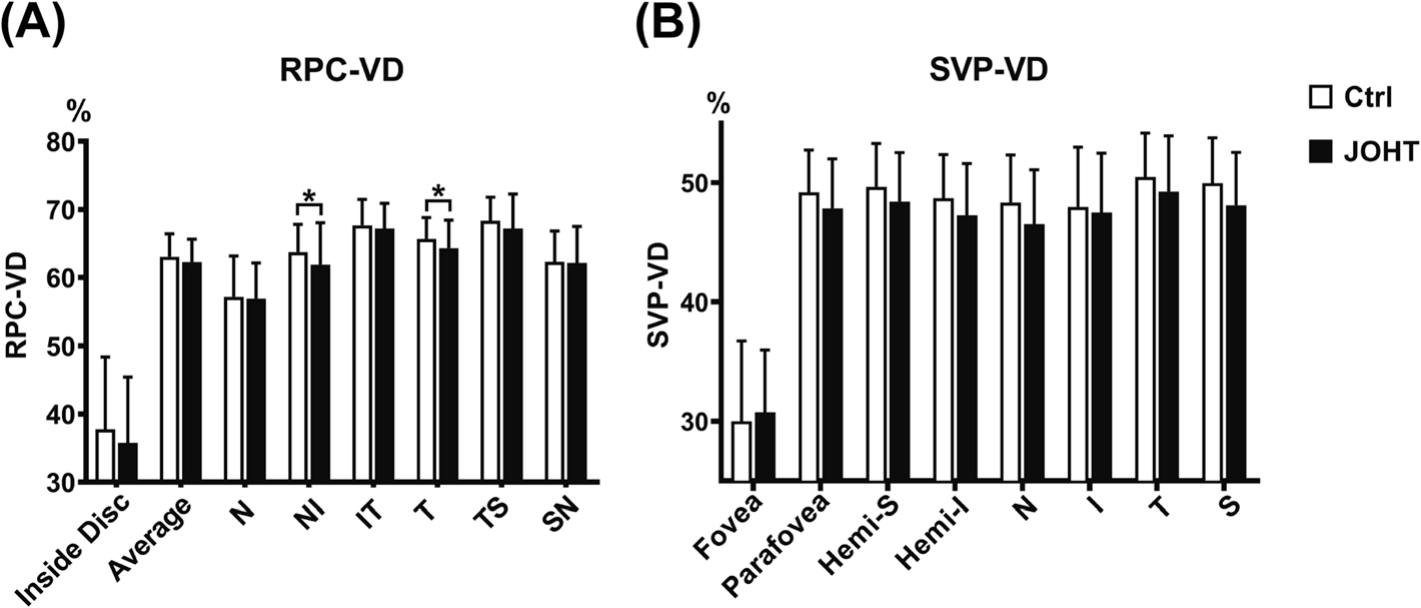
RPC-VD (**a**) and SVP-VD (**b**) of both Ctrl and JOHT subjects. Only NI and T of RPC-VD had significant differences between Ctrl and JOHT (*P* = 0.042 and *P* = 0.033; *P < 0.05). Data are shown as mean with error bars of standard deviation (SD). N, nasal; I, inferior; T, temporal; S, superior; RPC, radial peripapillary capillaries; SVP, supervisual vascular plexus; VD, vessel density; Ctrl, control; JOHT, juvenile ocular hypertension

A total of 86 eyes of 45 Ctrl subjects and 65 eyes of 34 JOHT subjects who initially met the eligibility criteria were included in the final analysis. The demographic and clinical characteristics of the two groups were summarized in Table 1. They were comparable for age, gender, SBP, DBP, MAP, PR, BCVA, CDR, GCC thickness, visual field MD, and PSD. Parameters of AL, CCT, IOP, and RNFL thickness were statistically different between Ctrl and JOHT subjects. Thus, IOP was further compared using a corrected formula as follows: Corrected IOP = Measured IOP - (CCT - 520) * 2.5/50 [25]. Corrected IOP of JOHT subjects was significantly higher than that of Ctrl as designed (*P* <  0.001). RPC-VD and SVP-VD were adjusted for AL, CCT, and RNFL differences in the post-hoc analysis to compare VD between JOHT and Ctrl. Bare differences were found in NI and T peripapillary regions (*P* = 0.042 and *P* = 0.033), while other regions showed no difference (Fig. 2).

**Table 1:** Demographic, clinical, and ocular characteristics of the Ctrl and JOHT subjects

	Ctrl eyes (86 eyes of 45 subjects)	JOHT eyes (65 eyes of 34 subjects)	*P* values
Demographic characteristics			
Age (years)^b^	28.2 ± 6.4	24.7 ± 12.0	0.127
Gender (male%)^c^	30%	50%	0.064
Clinical characteristics			
SBP (mmHg)^b^	118.8 ± 13.2	122.1 ± 19.4	0.421
DBP (mmHg)^a^	74.1 ± 9.8	74.1 ± 12.8	0.983
MAP (mmHg)^b^	89.0 ± 9.7	90.1 ± 14.2	0.724
PR (bpm)^a^	74.1 ± 14.2	78.5 ± 12.7	0.141
Ocular characteristics			
BCVA (logMAR)	1.0 ± 0.2	1.0 ± 0.1	0.840
AL (mm)	24.5 ± 1.1	24.9 ± 1.0	0.049
CCT (μm)	535.2 ± 33.7	565.2 ± 31.8	< 0.001
IOP (mmHg)	15.9 ± 2.3	24.9 ± 3.3	< 0.001
MOPP (mmHg)	43.4 ± 6.4	33.6 ± 10.2	< 0.001
Corrected IOP (mmHg)	15.2 ± 2.2	22.7 ± 4.5	< 0.001
CDR (vertical cup/disc)	0.6 ± 0.2	0.6 ± 0.1	0.998
RNFL (μm)	107.3 ± 9.4	104.0 ± 10.0	0.044
GCC (μm)	95.4 ± 6.6	93.2 ± 6.6	0.050
MD (dB)	−1.3 ± 1.1	− 1.5 ± 1.4	0.414
PSD (dB)	1.7 ± 0.5	1.9 ± 0.7	0.074

Unlabeled variables used a linear mixed model to adjust the eye-level factors

*Ctrl* control, *JOHT* juvenile ocular hypertension, *SBP* systolic blood pressure, *DBP* diastolic blood pressure, *MAP* mean arterial pressure, *PR* pulse rate, *BCVA* best-corrected visual acuity, *logMAR* logarithm of minimal angle resolution, *AL* axial length, *CCT* central corneal thickness, *IOP* intraocular pressure, *MOPP* mean ocular perfusion pressure, *CDR* cup/disc ratio, *RNFL* retinal nerve fiber layer, *GCC* ganglion cell complex, *MD* mean deviation, *PSD* pattern standard deviation

^a^Variables were analyzed using a two-sample t test due to normal distribution

^b^Variables were analyzed using the Kruskal-Wallis rank sum test due to non-normal distribution

^c^Variables of gender were compared using the chi-square test

All data from Ctrl and JOHT were included to evaluate the effect of IOP and CDR on RPC-VD (Table 2). An eye-gender, CCT, AL, and RNFL-adjusted linear mixed model showed that VD of the inside disc was significantly lower in subjects with CDR larger than 0.6 (r = − 13.304, *P* <  0.001; Table 2). RPC-VD of the T region was marginally negatively correlated with high IOP (r = − 1.329, *P* = 0.043; Table 2), while other regions showed no correlation with high IOP (all *P* > 0.05). Average RPC-VD, and N, IN, IT, ST, SN regions of RPC-VD were strongly correlated positively with CDR > 0.6 (r = 2.621, *P* <  0.001; r = 4.425, *P* <  0.001; r = 3.110, *P* <  0.001; r = 2.153, *P* = 0.001; r = 1.642, *P* = 0.019; r = 2.463, *P* = 0.006, respectively). Of the six peripapillary regions in RPC-VD, only the T region showed no correlation with CDR (r = 0.926, *P* = 0.152).

**Table 2:** Regression coefficient of IOP and CDR in assessment of VD

	r (IOP)	*P* values	r (CDR)	*P* values
Inside disc	−1.377	0.358	−13.304	< 0.001***
Fovea	0.895	0.466	0.942	0.418
RPC-VD				
Average	−0.887	0.122	2.621	< 0.001***
N	−0.599	0.545	4.425	< 0.001***
IN	−0.727	0.409	3.110	< 0.001***
IT	−1.240	0.074	2.153	0.001**
ST	−0.978	0.181	1.642	0.019*
SN	−0.598	0.520	2.463	0.006**
T	−1.379	0.043*	0.926	0.152
SVP-VD				
Parafovea	−1.530	0.041*	0.905	0.625
Hemi-S	−1.570	0.037*	0.950	0.180
Hemi-I	−1.449	0.064	0.936	0.204
T	−1.235	0.116	1.330	0.075
S	−1.877	0.023*	0.177	0.818
I	−1.236	0.209	1.389	0.137
N	−1.693	0.049*	0.883	0.276

Variables were analyzed using a linear mixed model with IOP > 21 mmHg or CDR > 0.6 defined as 1, while the opposite was defined as 0

*IOP* intraocular pressure, *CDR* cup/disc ratio, *VD* vessel density, *RPC* radial peripapillary capillaries, *SVP* superficial vascular plexus, *T* temporal, *I* inferior, *N* nasal, *S* superior

* *P* < 0.05; ** *P* < 0.01; *** *P* < 0.001

Linear mixed models adjusted by CCT, AL, RNFL, and eye gender also showed that perfusion of SVP had no correlation with large CDR, while high IOP did not (Table 2). S and N of SVP-VD were marginally lower in subjects with high IOP (r = − 1.877, *P* = 0.023; r = − 1.693, *P* = 0.049; Table 2). In addition, parafovea and hemi-S SVP-VD were found to be slightly statistically positively correlated with IOP more than 21 mmHg (r = − 1.530, *P* = 0.041; r = − 1.570, *P* = 0.037; Table 2). Unmentioned parameters were not found to be significant differences between the positive and negative values of IOP or CDR in SVP-VD (all *P* > 0.05, Table 2).

## Discussion

In this study, we reported the RPC-VD and SVP-VD between Ctrl and JOHT subjects. IN and T of RPC-VD were slightly higher in JOHT than in Ctrl subjects. Risk factors of CDR and IOP were further taken into consideration in the assessment of VD. A strong positive correlation between large CDR and RPC-VD in six regions except for T was established. The T region of RPC-VD had a tendency to decrease with IOP more than 21 mmHg, while its weak impact on S and N of SVP-VD was also observed. All of these data suggest that the results of OCTA scans showed the slight VD change in JOHT subjects. It also demonstrated strong correlation with CDR and exhibited useful advantages in long-term follow-up of JOHT.

The capillary blood flow of glaucoma patients, OHT, and Ctrl subjects has been evaluated using scanning laser Doppler flowmetry images in the past [26]. Subsequent research used OCTA to compare the similar parameters between Ctrl and OHT subjects and agreed with our first findings of no obvious alterations in SVP-VD (Fig. 2) [17, 23]. However, IN and T of RPC-VD showed statistical significance between Ctrl and JOHT, although the *p*-value was closed to 0.05. The average age of the subjects involved in previous studies was more than 40 years old, substantially different from the JOHT subjects we desired to target. Moreover, the CDR of the Ctrl they used accorded with the inclusion criteria of less than 0.4 by ophthalmoscope, while subjects of high CDR were sometimes considered glaucoma suspects or OHT when the IOP was above 21 mmHg. Regarding the wide range of CDR values in the normal population varying from 0.00 to 0.87, grouping was different from previous studies in our research. This contradiction made further analysis of IOP and CDR necessary; we thus applied a linear mixed model to analyze these two factors separately.

Using the linear mixed model to study the risk factor of IOP higher than 21 mmHg, T RPC-VD exhibited a significant reduction in JOHT subjects. Although no statistical correlation was found in the IT region, a consistent trend was observed throughout the comparison. Kerr and associates reported reduced blood flow in the temporal neuroretinal rim of the optic nerve; thus, the decrease of RPC-VD in our study respects the patterns of early glaucomatous changes [27]. Meanwhile, sectors of superior and nasal SVP-VD also exhibited decreases matching the patterns of peripapillary regions. It should be noted that we did not actually measure the blood flow of the retina using OCTA, but the imaging technique could be a useful tool to show the vascular changes by the index of VD. Hence, our results of reduction in both RPC and SVP-VD could be explained by a decrease in MOPP according to the formula which leads to a drop in ocular perfusion, as microvascular networks are of primary interest in glaucoma [20]. On the other hand, high-level evidence has shown that treatments decreasing IOP improve retinal circulation and reduce the risk of developing glaucoma in OHT [28]. This validates the effects of high IOP on ocular perfusion. Besides, individuals with high IOP are more likely to suffer from venous collapse, which then exacerbates impaired blood flow. OCTA proves the difference of capillary VD caused by high IOP in JOHT subjects and could serve as a precise method to monitor disease development, although longitudinal data are still needed to fully evaluate this possibility.

The RPC-VD of all six regions, except for the T sector, showed an increase with CDR > 0.6. No statistical difference was found in the perifoveal areas. CDR has been shown to be positively related to optic disk size, and the number of nerve fibers revealed consistency with the optic disk size, with the macula unaffected [29, 30]. RPC comprises a network of capillary beds located within the RNFL carrying metabolites to nerve fibers; thus, it should be correlated with the total number of nerve fibers. The positive relationship between CDR and RPC-VD in our study further clarifies this vascular structure of blood supply. However, the T sector showed no correlation with CDR. One reason for this could be that the T peripapillary region shares the thinnest RNFL according to ISNT rules. Taking application into consideration, CDR is used as an individual risk factor for OHT development with a wide normal range, which makes it confusing to distinguish large optic disc subjects with glaucoma suspects. OCTA quantifies these changes more precisely, which could aid in the evaluation of early perfusion alterations in JOHT subjects.

The main limitation of our study is the small sample size, especially JOHT subjects exhibiting both high IOP and normal CDR. Basically, our research is an exploratory study instead of a confirmative one. We wish to find some clues in OCTA scans of JOHT to provide any support for clinicians to pursuing further important results. The sample size applied in this study follows the previous publication of OCTA in Ctrl, OHT or glaucoma patients. The majority of the researchers conducted the research with 20–50 participants in each individual group. In future, we will evolve more participants in our study and pursue the long-time follow up to acquire more powerful evidence to support the findings. Additionally, IOP has always been normalized in the majority of juveniles over adolescence with long-term follow-up, which is why the medical terminology of ‘adolescence IOP fluctuation’ or ‘adolescence ocular hypertension’ is used. We only used IOP as a factor directly, and in the future we could test whether IOP alone or corrected IOP can exhibit more powerful correlations with the VD of JOHT subjects [31]. Besides, the software of OCTA applied in this study was not capable of removing the signals of large vessels. As explained above, more researches concerning disc size, CDR, and VD using the new upgraded device of OCTA are necessary. There is no long-term follow-up done to support the value of the clinical implications in OCTA of CDR or IOP [4]. In addition, we are still unclear about how IOP fluctuations accompany, for example, seasonal changes; this is a further target for the group to study.

## Conclusions

The current study demonstrated slightly lower nasal-inferior and temporal RPC-VD in JOHT subjects. A marginal negative relationship between T RPC-VD and high IOP was found, while CDR had the strong opposite outcomes. SVP-VD of perifoveal regions had a trend to reduce with high IOP but not by CDR. The results suggest that both the risk factor especially CDR should be considered and analyzed individually when clinicians assess the test results of OCTA scans in JOHT. RPC-VD, especially in the temporal region, has the potential value to assess the development of JOHT.

## Data Availability

The data sets used and/or analyzed during the current study are available from the corresponding author on reasonable request.

## Abbreviations

AL: Axial length
BCVA: Best-corrected visual acuity
BP: Blood pressure
Ctrl: Control subjects
CCT: Central corneal thickness
CDR: Cup/disc ratio
DBP: Diastolic blood pressure
EGPS: European glaucoma prevention study
GCC: Ganglion cell complex
I: Inferior
ILM: Inner limiting membrane
IOP: Intraocular hypertension
IPL: Inner plexiform layer
JOHT: Juvenile ocular hypertension
MAP: Mean arterial pressure
MD: Mean deviation
MOPP: Mean ocular perfusion pressure
N: Nasal
OCTA: Optical coherence tomography angiography
OHT: Ocular hypertension
OHTS: Ocular hypertension treatment study
PSD: Pattern standard deviation
RE: Refractive error
RGC: Retinal ganglion cell
RNFL: Retinal nerve fiber layer
RPC: Radial peripapillary capillaries
S: Superior
SAP: Standard automated perimetry
SBP: Systolic blood pressure
SD-OCT: Spectral-domain optical coherence tomography
SSADA: Split-spectrum amplitude-decorrelation angiography
SVP: Superficial vascular plexus
T: Temporal
VD: Vessel density
